# Elevation of Non-Classical (CD14^+/low^CD16^++^) Monocytes Is Associated with Increased Albuminuria and Urine TGF-β_1_ in HIV-Infected Individuals on Stable Antiretroviral Therapy

**DOI:** 10.1371/journal.pone.0153758

**Published:** 2016-04-20

**Authors:** Brooks I. Mitchell, Mary Margaret Byron, Roland C. Ng, Dominic C. Chow, Lishomwa C. Ndhlovu, Cecilia M. Shikuma

**Affiliations:** 1 Department of Tropical Medicine, University of Hawaii, Honolulu, Hawaii, United States of America; 2 Hawaii Center for AIDS, University of Hawaii, Honolulu, Hawaii, United States of America; 3 Department of Medicine, University of Hawaii, Honolulu, Hawaii, United States of America; COCHIN INSTITUTE, Institut National de la Santé et de la Recherche Médicale, FRANCE

## Abstract

**Objective:**

High rates of albuminuria are observed among HIV-infected individuals on stable antiretroviral therapy (ART). Though pro-inflammatory and pro-fibrotic responses are described as components of albuminuria in the general population, it is unclear how these responses are associated to albuminuria in ART-treated chronic HIV. We investigated the relationship of monocyte subsets and urine inflammatory and fibrotic biomarkers to albuminuria in ART-treated HIV-infected participants.

**Design and Methods:**

Cross-sectional analyses were performed on Hawaii Aging with HIV-cardiovascular disease study cohort participants who were required at entry to be ≥40 years old and on ART ≥3 months. Monocyte subpopulations were determined in banked peripheral blood mononuclear cells (PBMC) using multi-parametric flow-cytometry. Entry random urine samples were assessed for albumin-to-creatinine ratios (UACR). Urine samples were measured for inflammatory and fibrotic biomarkers using Luminex technology.

**Results:**

Among 96 HIV-infected subjects with measured UACR (87% male, 59% Caucasian, and 89% undetectable HIV RNA with median CD4 of 495.5 cells/μL), 18 patients (19%) had albuminuria. Non-classical (CD14^low/+^CD16^++^) monocytes were significantly elevated in subjects with albuminuria (p = 0.034) and were correlated to UACR (r = 0.238, p = 0.019). Elevated non-classical monocyte counts were significant predictors of worsening albuminuria, independent of traditional- and ART-associated risk factors (β = 0.539, p = 0.007). Urine TGF-β1 and collagen-IV were significantly higher in albuminuric compared to non-albuminuric participants (TGF-β1; p = 0.039 and collagen-IV; p = 0.042). Urine TGF-β1 was significantly correlated with non-classical monocyte counts (r = 0.464, p = 0.017).

**Conclusion:**

Alterations in monocyte subpopulations and urine pro-fibrotic factors may play a role in kidney dysfunction during chronic HIV infection and warrants further study.

## Introduction

Albuminuria is a strong and independent risk factor for renal and cardiovascular disease among the general and HIV-infected populations [1–7]. There is a higher prevalence of albuminuria observed among HIV-infected individuals (4–20%) as compared to those who are uninfected (2%), but mechanisms driving this remain poorly understood [5, 6, 8, 9]. Prior to the introduction of antiretroviral therapy (ART), albuminuria and other renal complications that occurred in individuals infected with HIV were commonly caused by HIV-associated nephropathy [10, 11]. With the widespread use of ART, etiologies of albuminuria have shifted to comorbid diseases such as hypertension and diabetes mellitus, as well as side effects of ART including Tenofovir [12–17]. Persistent pro-inflammatory and pro-fibrotic responses and immune dysfunction driven by chronic HIV infection during suppressive ART may also contribute to the prevalence of albuminuria in HIV-infected individuals [18–24].

Recently, monocytes have been implicated to play a role in the development of non-AIDS comorbidities during chronic HIV infection [25–30]. Through the advancement of flow-cytometry, various monocyte subpopulations have been phenotyped and are traditionally characterized into classical (CD14^++^CD16^-^), intermediate (CD14^++^CD16^+^), and non-classical (CD14^+/low^CD16^++^) subsets [31, 32]. We recently reported a novel CD14^+/low^CD16^-^ subpopulation termed transitional monocytes and described this subset to be associated with increased carotid intima media thickness in HIV-infected individuals [33]. Monocytes contribute to both the production of pro-inflammatory and pro-fibrotic cytokines, and may be major mediators of HIV-associated inflammation and fibrosis [34–38].

Utilizing clinical data and banked blood and urine specimens from a natural history study of individuals with chronic HIV on ART, we investigated the relationship of blood monocyte subsets and urine inflammatory and fibrotic biomarkers to albuminuria in ART-treated HIV-infected participants. We sought to assess the role of monocyte-associated inflammation and fibrosis in the pathogenesis of albuminuria during chronic HIV infection.

## Methods

### Study participants

This retrospective study was conducted utilizing entry data and specimens from the Hawaii Aging with HIV Cohort-Cardiovascular Disease (HAHC-CVD) study, a 5-year natural history study investigating the role of chronic HIV infection in the development of CVD among HIV-infected participants on suppressive ART. Inclusion criteria into the cohort required documented HIV-positive status, age ≥40 years, and use of combination ART ≥3 months. Extensive HIV immunologic and cardio-metabolic assessments were available from this cohort and further details have been previously described [39]. IRB approval was obtained from the University of Hawaii Human Studies Program (CHS #16476), and all participants provided written informed consent prior to enrollment into the HAHC-CVD study. Furthermore, at the time of entry into the cohort, all participants gave written informed consent to banking of specimens and use of clinical data and specimens for future studies related to HIV and its complications. All banked specimens and data collected from participants were anonymized and de-identified prior to analysis.

Random urine collections were obtained from participants upon entry into the HAHC-CVD study and urine albumin-to-creatinine ratios (UACR) were calculated. UACR was determined by immunotubidimetric assay using a Roche/Hitachi Modular P analyzer by a commercial College of American Pathologist (CAP)-certified laboratory (Diagnostic Laboratory Services Inc.). Albuminuria was defined as a UACR ≥30mg/g and was used to separate participants into 2 groups, with or without albuminuria.

### Peripheral blood mononuclear cell isolation and flow cytometry assay

Peripheral blood mononuclear cell (PBMC) isolation and flow cytometry assay was done as previously reported [30]. In brief, whole blood was drawn into EDTA tubes and cells were processed for PBMC isolation within one hour of collection and cryopreserved. Banked cryopreserved PBMCs were thawed and washed with warmed AIM-V liquid media (Invitrogen). Cells were then surface-stained with CD3, CD14, CD16, CD56, CD19, CD20, HLA-DR antibodies, and with Live/Dead fixable yellow dead cell stain (YARD). Data was acquired on a custom 4-laser BD LSRFortessa Cell Analyzer and all compensation and gating analyses were performed in FlowJo analytical software (gating strategy previously shown in figure 1 in reference [30]). Percentages of classical, intermediate, non-classical, and transitional monocyte subsets were determined based on CD14 and CD16 staining and absolute numbers of each subset were calculated from WBC and monocyte percent obtained from available clinical CBC performed on each participant.

### Detection of urine inflammatory and fibrotic biomarkers by Luminex

Random urine samples from participants collected at HAHC-CVD study entry were aliquoted and cryopreserved. Banked cryopreserved urine samples from randomly selected subjects demonstrating a range of UACR <30mg/g, in which all 78 participants were put into ascending order according to their measured UACR and every 4^th^ participant was chosen to be included in the urine biomarker assessment (for a total of 19), were tested along with all participants with a UACR ≥30mg/g (n = 18) for urinary inflammatory and fibrotic biomarkers. Cryopreserved urine aliquots were thawed and prepared following manufacturer’s guidelines for each kit. Each sample was measured in duplicate. Urine MCP-1 and IL-18 were measured using the Bio-Plex Pro™ RBM Human Kidney Toxicity Panel 1 (Bio-Rad). Urine TGF-β_1_, TGF-β_2_, and TGF-β_3_ were measured using the Bio-Plex Pro™ TGF-β Assay (Bio-RAD). Urine IP-10, Collagen IV, and TIMP-1 were measured using the Milliplex^®^ MAP Human Kidney Injury Magnetic Bead Panel 1 (EMD Millipore). Urine Cystatin C was measured using the Milliplex^®^ MAP Human Kidney Injury Magnetic Bead Panel 2 (EMD Millipore). Data was acquired on a Luminex 200™ analyzer (Luminex) and data analysis was done using Bio-Plex Manager™ software (Bio-Rad). Net median fluorescent intensity (MFI) was calculated (MFI value minus background value) and average net MFI of duplicate samples was determined.

### Statistical analysis

Comparisons of clinical and laboratory characteristics between groups were calculated using Mann-Whitney U and Chi-squared tests for continuous and categorical variables, respectively. UACR, absolute counts of cellular immune parameters, and net MFI averages of urine biomarkers were all log-transformed prior to correlation and linear regression analyses to attain normal distribution. Pearson product-moment correlation and multivariable linear regression were utilized to assess associations. In assessing the relationship between cellular immune parameters and UACR, cellular compartments with a Pearson product-moment correlation significant at p <0.05 were further examined in a separate multivariable linear regression model with UACR as the dependent variable, adjusting for age, hypertension, HOMA-IR, total cholesterol/HDL cholesterol ratio, and current use of Tenofovir and/or Ritonavir. A two-sided probability of *p*-value <0.05 was considered statistically significant. Statistical analyses were performed using the SPSS statistical program (SPSS Statistics 22, Armonk, NY).

## Results

### Characteristics of participants

On baseline evaluation, 96 HIV-infected participants on stable ART with UACR and monocyte subset analyses were available. Of these participants, 18 (19%) had albuminuria with the majority [16 participants (89%)] assessed to have moderately increased albuminuria (UACR 30-300mg/g) with only 2 (11%) having severe albuminuria (UACR >300mg/g). Demographics and clinical parameters are summarized in Table 1. Rates of viral suppression and CD4 T cell counts were comparable between the HIV-infected groups with or without albuminuria. HIV-infected participants with albuminuria were older, had higher rates of hypertension and use of angiotensin-converting enzyme (ACE) inhibitors and angiotensin II receptor blockers (ARB), had higher blood glucose levels at 120 minutes during oral glucose tolerance test (OGTT), and had higher insulin resistance measured by homeostatic model assessment-insulin resistance (HOMA-IR); but did not differ in rates of type 2 diabetes mellitus or use of Tenofovir or Ritonavir.

**Table 1:** Comparison of demographic and clinical parameters of HIV-infected participants on ART with and without albuminuria^(^^a^^)^.

Demographic and Clinical Parameters			
Parameter	Patients with albuminuria	Patients without albuminuria	p-value
n = 96	n = 18	n = 78	
Age, years	57 [49, 62.5]	50 [45, 55]	0.013*
Male, n (%)	16 (88.9%)	67 (86.0%)	1.000
Race, n (%)			
Caucasian	12 (66.7%)	45 (57.7%)	0.665
Other races	6 (33.3%)	33 (42.3%)	0.665
History of smoking, n (%)	12 (66.7%)	49 (63.6%)	1.000
Body mass index, kg/m^2^	25.9 [21.8, 28.1]	25.7 [23.8, 27.9]	0.789
Systolic blood pressure, mmHg	124.5 [119.8, 141.3]	120.0 [111.3, 129.0]	0.043*
Diastolic blood pressure, mmHg	80.5 [75.0, 84.3]	74.0 [68.0, 80.0]	0.012*
History of hypertension, n (%)	12 (66.7%)	24 (30.8%)	0.010*
Current use of ACE inhibitors and/or ARB ^(^^b^^)^, n (%)	9 (50%)	12 (15%)	0.004**
Fasting glucose, mg/dL	91.5 [80.5, 96.75]	87.0 [81.0, 94.0]	0.366
Fasting insulin, μIU/mL	9.4 [4.7, 13.5]	5.9 [3.65, 10.1]	0.060
OGTT ^(^^c^^)^ blood glucose @ 120 min, mg/dL	113.0 [96.0, 138.0]	97.5 [73.3, 115.5]	0.031*
Metabolic syndrome ^(^^d^^)^, n (%)	5 (27.8%)	15 (19.2%)	0.629
Type 2 diabetes mellitus, n (%)	3 (16.7%)	7 (9.0%)	0.593
HOMA-IR ^(^^e^^)^	2.6 [1.2, 3.4]	1.2 [0.8, 2.2]	0.017*
eGFR CKD-EPI ^(^^f^^)^, mL/min/1.73 m^2^	72.8 [63.8, 101.1]	86.6 [76.2, 99.3]	0.071
UACR ^(^^g^^)^, mg/g	63.35 [44.40, 156.15]	4.95 [3.38, 8.92]	<0.001**
Blood Urea Nitrogen, mg/dL	13.5 [11.0, 18.0]	13.0 [10.0, 16.3]	0.327
Serum creatinine, mg/dL	1.1 [0.9, 1.3]	1.0 [0.9, 1.1]	0.168
Serum cystatin C, mg/L	0.80 [0.72, 0.90]	0.74 [0.67, 0.82]	0.137
Total cholesterol, mg/dL	180.0 [139.8, 267.0]	174.5 [150.8, 192.3]	0.383
LDL cholesterol, mg/dL	119.0 [75.3, 180.5]	104.5 [83.8, 124.3]	0.315
HDL cholesterol, mg/dL	36.0 [31.0, 55.3]	41.0 [32.5, 49.0]	0.683
Triglycerides, mg/dL	116.5 [92.8, 171.8]	109.5 [75.8, 161.5]	0.406
Total cholesterol/HDL ratio	4.14 [3.48, 6.58]	4.09 [3.46, 5.29]	0.546
HIV RNA < 50 copies/mL, n (%)	16 (88.9%)	68 (87.2%)	1.000
Current use of Tenofovir, n (%)	16 (88.9%)	58 (74.3%)	0.312
Current use of Ritonavir, n (%)	7 (38.9%)	29 (37.2%)	1.000

a. Median values shown with [median Q1, median Q3] or frequency, n with (%).

*p <0.05

**p <0.01

b. Angiotensin-Converting Enzyme Inhibitor and Angiotensin II Receptor Blocker

c. Oral Glucose Tolerance Test

d. Metabolic syndrome was defined as having 3 or more of the following: Abnormal obesity, high triglycerides, low HDL cholesterol, high blood pressure, or high fasting glucose.

e. Homeostatic Model Assessment of Insulin Resistance was calculated by: HOMA-IR = [(fasting glucose/18) × fasting insulin]/22.5

f. Estimated Glomerular Filtration Rate was calculated using the CKD-EPI (Chronic Kidney Disease Epidemiology Collaboration) formula: eGFR (if serum creatinine ≤ 0.9 mg/dL) = 141 x (Serum Creatinine (mg/dL)/0.9)^-0.411^ x 0.993^Age^; eGFR (if serum creatinine > 0.9 mg/dL) = 141 x (Serum Creatinine (mg/dL)/0.9)^-1.209^ x 0.993^Age^

g. Urine Albumin/Creatinine Ratio was calculated by: UACR (mg/g) = Urine Albumin (mg/dL)/Urine Creatinine (g/dL) ≈ Albumin excretion in mg/day

### Elevated non-classical (CD14^+/low^CD16^++^) monocytes are associated with albuminuria in treated HIV-infected participants

T cell and monocyte subset percentages and counts were compared between participants with and without albuminuria. Non-classical monocyte counts were significantly elevated in HIV-infected participants with albuminuria as compared to HIV-infected participants without albuminuria (Table 2). No significant differences were seen in the T cell subsets counts or in the other monocyte subsets counts. Of the cellular parameters assessed, UACR significantly correlated with non-classical monocyte counts (r = 0.238, *p* <0.05), but not with CD4 T cells (r = 0.070), CD8 T cells (r = 0.150), activated CD8 T cells (r = -0.039), or with classical (r = 0.112), intermediate (r = 0.067), or transitional monocytes (r = 0.127).

**Table 2:** Comparison of immunological parameters of HIV-infected participants on ART with and without albuminuria^(^^a^^)^.

Immunological Parameters			
Parameter	Patients with albuminuria	Patients without albuminuria	p-value
n = 96	n = 18	n = 78	
Nadir CD4^+^ T cells, cells/μL	92.0 [33.0, 198.25]	170.0 [39.5, 249.5]	0.277
CD4^+^ T cells, cells/μL	496.50 [374.5, 554.5]	495.50 [329.75, 635.75]	0.767
CD4^+^ T cells, %	24.0 [21.0, 35.0]	30.0 [22.0, 37.0]	0.247
CD8^+^ T cells, cells/μL	885.5 [533.0, 1160.0]	693.0 [552.0, 946.5]	0.177
CD8^+^ T cells, %	49.0 [44.5, 54.3]	43.0 [34.5, 50.0]	0.070
Activated CD8^+^ T cells (CD38^+^), cells/μL	73.2 [53.7, 127.7]	77.8 [46.6, 123.8]	0.962
Activated CD8^+^ T cells (CD38^+^), %	11.2 [7.7, 14.0]	11.0 [7.9, 16.8]	0.584
Total monocytes, cells/L	4.5x10^8^ [3.7x10^8^, 6.1x10^8^]	3.9x10^8^ [3.2x10^8^, 5.0x10^8^]	0.070
Total monocytes, %	8.0 [7.0, 10.0]	8.0 [7.0, 9.0]	0.875
Classical (CD14^++^CD16^-^) monocytes, cells/L	3.4x10^8^ [2.6x10^8^, 4.8x10^8^]	3.0x10^8^ [2.2x10^8^, 4.0x10^8^]	0.110
Classical (CD14^++^CD16^-^) monocytes, %	75.4 [68.0, 80.6]	75.9 [70.3, 81.2]	0.645
Intermediate (CD14^++^CD16^+^) monocytes, cells/L	8.2x10^6^ [2.3x10^6^, 1.5x10^7^]	5.0x10^6^ [2.3x10^6^, 1.2x10^7^]	0.344
Intermediate (CD14^++^CD16^+^) monocytes, %	1.3 [0.5, 3.9]	1.2 [0.5, 2.8]	0.820
Non-classical (CD14^+/low^CD16^++^) monocytes, cells/L	2.7x10^7^ [2.0x10^7^, 5.5x10^7^]	2.0x10^7^ [1.3x10^7^, 3.0x10^7^]	0.033*
Non-classical (CD14^+/low^CD16^++^) monocytes, %	7.0 [4.0, 8.9]	5.0 [3.7, 8.4]	0.226
Transitional (CD14^+^CD16^-^) monocytes, cells/L	6.8x10^7^ [5.2x10^7^, 1.1x10^8^]	5.8x10^7^ [4.5x10^7^, 8.2x10^7^]	0.116
Transitional (CD14^+^CD16^-^) monocytes, %	15.3 [10.6, 19.6]	15.1 [10.6, 20.2]	0.777

a. Median values shown with [median Q1, median Q3]

*p <0.05

We assessed the predictive value of non-classical monocytes in a multivariable linear regression model, adjusting for traditional risk factors of age, hypertension, HOMA-IR, total cholesterol/HDL cholesterol ratio, and ART-associated risk factors of current use of Tenofovir and/or Ritonavir (Table 3). Univariable linear regression analyses of risk factors predicting UACR are as follows: Age, B = 0.017, *p* = 0.035, C.I. = 0.001–0.033; Hypertension, B = 0.463, *p*<0.001, C.I. = 0.235–0.691; HOMA-IR, B = 0.077, *p* = 0.010, C.I. = 0.019–0.136; Total cholesterol/HDL cholesterol ratio, B = 0.067, *p* = 0.046, C.I. = 0.001–0.132; Current use of Tenofovir, B = 0.215, *p* = 0.131, C.I. = -0.066–0.496; Current use of Ritonavir, B = -0.027, *p* = 0.827, C.I. = -0.274–0.220. We conclude that elevation of non-classical (CD14^low/+^CD16^++^) monocytes significantly predict worsening albuminuria in HIV-infected participants on stable ART, independent of traditional- and ART-associated risk factors.

**Table 3:** Multivariable linear regression analysis^(a)^ of non-classical (CD14^+/low^CD16^++^) monocyte counts as a predictor of albuminuria in HIV-infected participants on ART while adjusting for risk factors (n = 96).

	Unstandardized Coefficients		Standardized β-value	*p*-value	95% C.I. for B-value	
	B-value	Std. Error			Lower	Upper
Non-classical Monocytes	0.539	0.197	0.259	0.007**	0.148	0.930
Age	0.017	0.008	0.212	0.038*	0.001	0.034
Hypertension	0.260	0.123	0.213	0.037*	0.017	0.504
HOMA-IR	0.026	0.028	0.234	0.017*	0.012	0.125
Total Cholesterol/HDL ratio	0.027	0.030	0.084	0.366	-0.032	0.087
Current use of Tenofovir	0.357	0.132	0.259	0.008**	0.094	0.621
Current use of Ritonavir	0.024	0.113	0.020	0.834	-0.201	0.248

a. The dependent variable for the performed multivariable linear regression model was urine albumin/creatinine ratio (UACR) and was log-transformed prior to analysis. The non-classical monocyte subset count was log-transformed prior to analysis and was inputted as a predictor variable for albuminuria to assess its significance in a multivariable model.

*p <0.05

**p <0.01

### Elevated non-classical (CD14^+/low^CD16^++^) monocytes are associated with elevated urine TGF-β_1_ in ART-treated HIV-infected participants

Urinary inflammatory and fibrotic biomarkers were assessed in all 18 participants with albuminuria and in 19 randomly selected participants out of the total of 78 participants without albuminuria. Between the 19 participants selected for urine biomarker analyses and the 59 participants that were not selected, there were no statistical differences observed in demographic, clinical, or immunological parameters measured including age, history of hypertension, HOMA-IR, and UACR. HIV-infected participants with albuminuria had significantly higher urinary levels of fibrotic markers TGF-β_1_ and collagen IV (Table 4). There were no significant differences observed in measured urine inflammatory biomarkers between groups.

**Table 4:** Comparison of measured urine biomarkers of HIV-infected participants on ART with and without albuminuria^(^^a^^)^.

Urine Biomarkers			
Biomarker^(^^a^^)^	Patients with albuminuria	Patients without albuminuria	p-value
n = 37	n = 18	n = 19	
MCP-1^(^^b^^)^	80.0 [22.3, 178.9]	132.0 [7.3, 214.8]	0.663
IP-10^(^^c^^)^	5.3 [0.4, 17.3]	3.0 [0, 14.8]	0.274
IL-18^(^^d^^)^	4.8 [0.9, 17.4]	8.5 [0, 19.0]	0.604
Cystatin C	88.0 [19.9, 240.4]	68.0 [6.8, 125.3]	0.181
TGF-β_1_^(^^e^^)^	14.4 [5.8, 33.8]	3.5 [0, 11.5]	0.039*
TGF-β_2_	12.8 [5.1, 20.5]	7.5 [5.0, 21.3]	0.503
TGF-β_3_	8.1 [6.9, 22.1]	7.3 [3.3, 17.3]	0.152
Collagen IV	1160.9 [565.9, 1470.1]	702.0 [344.5, 888.5]	0.042*
TIMP-1^(^^f^^)^	678.8 [235.3, 1420.2]	516.5 [171.5, 751.3]	0.178

a. Medians of net median fluorescence intensity (MFI) averages are shown with [median Q1, median Q3].

*p <0.05

b. Monocyte Chemotactic Protein-1 (CCL2)

c. IFN-γ-Inducible Protein 10 (CXCL10)

d. Interleukin-18

e. Tumor Growth Factor-β

f. Tissue Inhibitor of Metalloproteinase-1

We assessed the correlation between the measured urine inflammatory and fibrotic biomarkers with non-classical monocytes. Urine TGF-β_1_ was significantly correlated with non-classical monocytes (r = 0.464, *p* <0.05), while IP-10 (r = 0.008), MCP-1 (r = -0.141), IL-18 (r = -0.037), Cystatin C (r = -0.129), TGF-β_2_ (r = 0.063), TGF-β_3_ (r = -0.189), collagen IV (r = 0.032), and TIMP-1 (r = 0.029) did not show significant correlations. No correlations were seen between other monocyte subsets or T cell subpopulations and urine inflammatory or fibrotic biomarkers. We also assessed the correlations among the urine inflammatory and fibrotic biomarkers. TGF-β_1_ strongly correlated with TGF-β_2_ (r = 0.752) and TIMP-1 (r = 0.424). Furthermore, TGF-β_2_ correlated with TGF-β_3_ (r = 0.389), collagen IV (r = 0.617), and TIMP-1 (r = 0.730). Of the urine pro-inflammatory biomarkers, only urine MCP-1 correlated with TGF-β_2_ (r = 0.520), TGF-β_3_ (r = 0.362), collagen IV (r = 0.558), and TIMP-1 (r = 0.592).

## Discussion

Our study is the first report showing that albuminuria in HIV-infected individuals on stable ART is associated with increased levels of non-classical (CD14^low/+^CD16^++^) monocytes, independent of traditional and ART-associated risk factors. Treated HIV-infected individuals with albuminuria excrete higher levels of urine TGF-β1 and collagen IV as compared to those without albuminuria, and increased non-classical monocytes are associated with increased urine TGF-β1. Furthermore, urinary levels of TGF-β1 are strongly associated with other urine fibrotic markers. It is important to note that although only 89% of the participants were virally suppressed, when participants with viral loads were excluded, all significant observations stated remained significant.

HIV-infected individuals on stable ART show higher rates of albuminuria, as compared to the general population. Chronic low-grade inflammation may contribute to the development of albuminuria and renal dysfunction, which both pro-inflammatory and pro-fibrotic responses are important components [18, 20, 40–46]. However, mechanisms of albuminuria that occurs during ART-treated chronic HIV infection have been primarily focused on the pro-inflammatory arm. Results of our present study suggest a dynamic interplay between pro-inflammatory and pro-fibrotic responses.

CD16^+^ monocyte compartment, which include non-classical (CD14^low/+^CD16^++^) and intermediate (CD14^++^CD16^+^) subsets, are generally characterized as a pro-inflammatory cellular compartment, being potent producers of TNF-α, IL-6, IL-1β and IL-12 [47–51]. Several studies have shown that the recruitment of CD16^+^ monocytes in human inflammatory disease states may mediate further pro-inflammatory responses. Individuals with rheumatoid arthritis have been shown to have significantly higher CD16^+^ monocytes as compared to healthy controls [52, 53]. Elevation of this monocyte compartment is associated with worsening disease state, higher erythrocyte sedimentation rates, and increased C-reactive protein and rheumatoid factor levels. In respect to kidneys, non-classical monocytes have been shown to accumulate in the glomerular vessels and play a role in the development of lupus-associated glomerulonephritis [34, 54]. Non-classical monocytes were observed to preferentially produce TNF and CCL3 in serum from individuals with lupus.

Elevated levels of peripheral non-classical monocytes have also been described in HIV-infected individuals as compared to HIV-uninfected controls [26, 55, 56]. In addition, we have previously reported increased percentages of total monocytes producing pro-inflammatory cytokines IL-1β and IL-8 in ART-treated HIV-infected individuals as compared to HIV-uninfected individuals at basal levels and after stimulation with oxidized low-density lipoproteins and lipopolysaccharides [35]. With these findings, we suspect that the low-level chronic inflammatory environment that has been characterized in chronic HIV infection may be contributing to elevated levels of non-classical monocytes in peripheral blood and an active inflammatory phenotype that may contribute to the development of albuminuria [22].

Contrasting studies in the general population have shown non-classical monocytes in humans to play a role in the resolution of inflammation and the differentiation into M2 macrophages that aide in anti-inflammatory and wound healing responses [57]. In myocardial infarctions, non-classical monocytes have been shown to demonstrate a beneficial effect in mediating vascular repair and organ function [58–60]. Similarly, recruitment of non-classical monocytes into the brain and spinal cord have shown to be associated with beneficial effects, which include active removal of amyloid-β peptides in neuronal tissue and maintenance of the blood-brain barrier by differentiated perivascular macrophages [61–63].

During the resolution of inflammation, an important component of the response is TGF-β. TGF-β is a multi-functional cytokine that regulates many cellular functions, including cellular growth and differentiation [64]. In the context of HIV infection, TGF-β is observed to be elevated in infected individuals and has been suggested to contribute to the pathogenesis of HIV-associated nephropathy [23, 24, 65]. Mesangial cells have been reported to contribute to the elevated production of TGF-β in the kidneys of HIV-infected individuals [23, 24]. Peripheral blood mononuclear cells have also been found to produce and secrete TGF-β, with associated elevation of TGF-β in the plasma and tissues [37, 38, 66–69]. Thus, monocytes/macrophages infected with HIV may also contribute to the local production of TGF-β in the kidneys. As our results show, non-classical monocytes are associated with increase intra-renal production of TGF-β as measured in urine. This suggests that in chronic HIV infection, monocyte-derived TGF-β may be increased and contribute to the elevated intrarenal levels, further driving albuminuria. Additional studies are warranted to assess these mechanisms.

This study is limited by its relatively small sample size and the lack of HIV-uninfected controls with measured UACR and phenotyped immunologic cellular subpopulations. However, the strengths of the study are the careful clinical and cardio-metabolic characterizations performed on HIV-infected groups, as well as detailed phenotypes of T cell and monocyte subpopulations and quantification of urine biomarkers that reveal discriminating associations in the HIV-infected group.

In conclusion, elevation of non-classical (CD14^low/+^CD16^++^) monocytes is associated with worsening albuminuria in HIV-infected individuals on ART. This association is independent of traditional- and ART-associated risk factors of albuminuria. Furthermore, HIV-infected individuals with albuminuria excrete higher levels of urine TGF-β1 and collagen IV as compared to those without albuminuria, and increased non-classical monocytes are associated with increased urine TGF-β1. The role of non-classical monocytes in pro-inflammatory and/or pro-fibrotic responses in the kidney of HIV-infected individuals on ART warrants further study.
